# Development and Validation of a Recurrence-Free Survival Prediction Model for Locally Advanced Esophageal Squamous Cell Carcinoma with Neoadjuvant Chemoradiotherapy

**DOI:** 10.1245/s10434-023-14308-3

**Published:** 2023-09-26

**Authors:** Yehan Zhou, Wenwu He, Peng Guo, Chengmin Zhou, Min Luo, Ying Liu, Hong Yang, Sheng Qin, Xuefeng Leng, Zongyao Huang, Yang Liu

**Affiliations:** 1 Present Address: Department of Pathology, Sichuan Cancer Hospital and Institute, Sichuan Cancer Center, School of Medicine, University of Electronic Science and Technology of China, Chengdu, China; 2 Department of Thoracic Surgery, Sichuan Cancer Hospital and Institute, Sichuan Cancer Center, School of Medicine, University of Electronic Science and Technology of China, Chengdu, China; 3 School of Medicine, University of Electronic Science and Technology of China, Chengdu, China; 4 Graduate School, Chengdu Medical College, Chengdu, China

**Keywords:** Nomograms, Neoadjuvant chemoradiotherapy, Recurrence-free survival, Esophageal squamous cell carcinoma

## Abstract

**Background:**

A recurrence-free survival (RFS) prediction model was developed and validated for patients with locally advanced esophageal squamous cell carcinoma treated with neoadjuvant chemoradiotherapy (NCRT) in combination with surgery.

**Patients and Methods:**

We included 282 patients with esophageal squamous cell carcinoma who received neoadjuvant chemoradiotherapy (NCRT) combined with surgery, constructed three models incorporating pathological factors, investigated the discrimination and calibration of each model, and compared the clinical utility of each model using the net reclassification index (NRI) and the integrated discrimination index (IDI).

**Results:**

Multivariable analysis showed that pathologic complete response (pCR) and lymph node tumor regression grading (LN–TRG) (*p* < 0.05) were independent prognostic factors for RFS. LASSO regression screened six correlates of LN-TRG, vascular invasion, nerve invasion, degree of differentiation, platelet grade, and a total diameter of residual cancer in lymph nodes to build model three, which was consistent in terms of efficacy in the training set and validation set. Kaplan–Meier (K–M) curves showed that all three models were able to distinguish well between high- and low-risk groups (*p* < 0.01). The NRI and IDI showed that the clinical utility of model 2 was slightly better than that of model 1 (*p* > 0.05), and model 3 was significantly better than that of model 2 (*p* < 0.05).

**Conclusions:**

Clinical prediction models incorporating LN-TRG factors have high predictive efficacy, can help identify patients at high risk of recurrence after neoadjuvant therapy, and can be used as a supplement to the  AJCC/TNM staging system while offering a scientific rationale for early postoperative intervention.

**Supplementary Information:**

The online version contains supplementary material available at 10.1245/s10434-023-14308-3.

Esophageal cancer ranks fourth in terms of cancer mortality in China, with half of all cases occurring within the country.^1^ The majority of these cases are squamous cell carcinomas that have reached advanced stages,^2^ and neoadjuvant therapy combined with surgery has been shown to significantly improve prognosis compared with surgery alone.^3^ Based on studies such as NEOCRTEC5010 and CROSS, neoadjuvant chemoradiotherapy (NCRT) has become the standard neoadjuvant therapy for locally advanced esophageal cancer, with pathologic complete response (pCR) rates reaching up to 49%.^4–6^ However, the long-term survival outcome is suboptimal with a maximum 5 year survival rate of less than 50%, and tumor recurrence remains the leading cause of mortality.^7^ CheckMate-577 and numerous other studies have demonstrated that postoperative adjuvant therapy can effectively reduce recurrence in high-risk patients.^8,9^ Therefore, predicting the risk of tumor recurrence has become an urgent issue to be addressed.

The implementation of adjuvant therapy has resulted in alterations to the condition of tumor cells. This has made the previous pathological AJCC/TMN staging system, which solely evaluates tumor depth and distribution, less proficient in determining prognosis.^9^ To address this issue, a grading system called the pathological tumor regression grade (TRG) has been developed. This system evaluates the number and proportion of residual tumors, the status of tumor cells, and the amount and extent of fibrosis after treatment-induced tumor regression, and assigns a grade based on these indicators. TRG is a reliable predictor of short-term efficacy and is widely used to assess treatment response at the primary site following neoadjuvant therapy for tumors.^10^ However, some researchers have found that the degree of response in the primary site and lymph nodes is not consistent,^11,12^ but to date, there is no internationally accepted standard grading system for assessing the treatment response of lymph nodes. In addition to the status of the tumor itself, the patient’s systemic status (routine blood tests and tumor markers checked weekly during neoadjuvant therapy), such as immunoinflammatory-related indices, has also been shown to correlate with prognosis,^13^ and studies have also shown that lower platelet, white blood cell, and hemoglobin scores are associated with a poorer prognosis (*p* < 0.001).^14^ Therefore, a comprehensive evaluation of multiple clinical and pathological dimensions is necessary for the accurate prediction of tumor recurrence risk. One effective approach to achieving this goal is through the establishment of a prediction model. However, there currently exists no reliable model for predicting recurrence-free survival (RFS) in patients who have undergone NCRT.

Therefore, this study aims to develop a new prediction model to predict the risk of recurrence and to complement the AJCC/TNM staging system, as well as to provide a research basis for early adjuvant therapy.

## Patients and Methods

### Research Objectives

The study population was obtained from surgically resected specimens of esophageal squamous cell carcinoma admitted to Sichuan Cancer Hospital from June 2017 to June 2021. Patient inclusion and exclusion criteria were as follows: (1) primary locally advanced esophageal squamous cell carcinoma, (2) no other combined malignancies, (3) preoperative concurrent neoadjuvant chemoradiotherapy, (4) radical esophageal cancer resection after neoadjuvant treatment, (5) no basis for distant metastasis.

A total of 282 patients were enrolled in the study and received preoperative neoadjuvant chemoradiotherapy with single or double combinations of albumin-bound paclitaxel, carboplatin, cisplatin, docetaxel, oxaliplatin, fluorouracil, tegafur, gimeracil, and oteracil potassium, with 1.99 ± 0.52 chemotherapy sessions, and radiotherapy targeting the primary tumor, positive lymph nodes, subclinical lesions, and lymph node drainage areas using image-guided intensity-modulated radiation therapy (IMRT) with split doses: gross tumor volume (GTV) 2.0 Gy/f, gross tumor volume lymph node left (GTVInL) 2.0 Gy/f, gross tumor volume lymph node right (GTVInR) 2.0 Gy/f, CTV (clinical target volume) 1.8 Gy/f, 20.32 ± 1.14 fractions, total dose 40.49 ± 1.75 Gy.

### Specimens Processing and Grading Criteria

Specimens were fixed in 10% neutral formalin, embedded in paraffin, routinely sectioned, and hematoxylin–eosin stained. Immunohistochemistry was performed by the streptavidin–peroxidase (SP) method, and the staining procedure was performed on a fully automated immunohistochemistry instrument according to the operating instructions. Primary tumor regression grading (PT–TRG) uses Becker grading criteria and is divided into four grades based on the percentage of live tumor cells remaining (grade 1: no residual tumor, grade 2: less than 10% residual tumor, grade 3: 10–50% residual tumor, grade 4: greater than 50% residual tumor),^15^ and lymph node tumor regression grading (LN–TRG) is divided into five grades (grade 0: true negative without any basis for tumor residual; grade 1: 0% residual lymph node cancer; grade 2: less than 10% residual lymph node cancer; grade 3: 10–50% residual lymph node cancer; grade 4: greater than 50% residual lymph node cancer). The total diameter of residual lymph node cancer was the sum of the largest diameter of living tumors in all lymph nodes (Supplementary Fig. 1). Leukocytes, hemoglobin, neutrophils, platelets, and myelosuppression grade refer to Common Terminology Criteria Adverse Events in Oncology 4.03 (CTCAE) (Supplementary Table 1)

### Data Statistics

One-way Cox regression, Kaplan–Meier curves, and log-rank tests were used for recurrence-free survival analysis, and Cox regression was used for multivariable analysis. The data set was divided into a training set and validation set by 5:5 random sampling, and the variables were screened using least absolute shrinkage and selection operator (LASSO) regression analysis, incorporated into multivariable Cox regression analysis, and constructed prediction models. Receiver operating characteristic (ROC) curves and calibration curves were used to assess model efficacy, and all analyses were performed using R statistical software (http://www.R-project.org, The R Foundation) and the Free Statistics analysis platform. Recurrence-free survival (RFS) was defined as the time interval from the date of surgery to recurrence or death from any cause. A *p*-value < 0.05 was considered statistically significant.

## Results

Table 1 presents the descriptive data: the follow-up time for cases was 25.18 ± 22.84 months and the clinical characteristics of 282 patients were as follows: ≤ 65 years old (68.8%), male (84.8%), smoker (68.1%), alcohol drinkers (66.3%), tumors with poor to middle differentiation (91.5%), located in the low and middle segment (86.2%), and R0 resection (98.6%). Of these, 90 (31.9%) cases reached pCR, 96 (34%) cases had a relapse (locoregional recurrence, distant metastases), with a mean relapse time of 16.61 ± 10.53 months, and 89 (92.7%) of the relapses occurred within 3 years. Cases were divided into training and validation sets by a 5:5 random sample, and there were no statistical differences between the two groups in all indicators except pCR (*p* = 0.041) (Table 1).

**Table 1:** Clinical and pathological characteristics of the training cohort and internal validation

Variables	Total (*n* = 282)	Training cohort (*n* = 141)	Internal validation (*n* = 141)	*p*-Value	Statistic
Sex, *n* (%)				0.408	0.686
Male	239 (84.8)	122 (86.5)	117 (83)		
Female	43 (15.2)	19 (13.5)	24 (17)		
Age, *n* (%)				0.999	0
< 65 years	194 (68.8)	97 (68.8)	97 (68.8)		
≥ 65 years	88 (31.2)	44 (31.2)	44 (31.2)		
Smoker, *n* (%)				0.201	1.632
No	90 (31.9)	40 (28.4)	50 (35.5)		
Yes	192 (68.1)	101 (71.6)	91 (64.5)		
Drinker, *n* (%)				0.059	3.572
No	95 (33.7)	40 (28.4)	55 (39)		
Yes	187 (66.3)	101 (71.6)	86 (61)		
Location, *n* (%)				0.468	1.52
Up	39 (13.8)	18 (12.8)	21 (14.9)		
Middle	108 (38.3)	59 (41.8)	49 (34.8)		
Low	135 (47.9)	64 (45.4)	71 (50.4)		
Differentiation, *n* (%)				0.495	1.408
Well	24 (8.5)	14 (9.9)	10 (7.1)		
Middle	158 (56.0)	81 (57.4)	77 (54.6)		
Poor	100 (35.5)	46 (32.6)	54 (38.3)		
Surgical margin, *n* (%)				0.122	Fisher
Negative	278 (98.6)	137 (97.2)	141 (100)		
Positive	4 (1.4)	4 (2.8)	0 (0)		
Nerve invasion, *n* (%)				0.424	0.638
Negative	235 (83.3)	115 (81.6)	120 (85.1)		
Positive	47 (16.7)	26 (18.4)	21 (14.9)		
Lymph, vascular invasion, *n* (%)				0.999	0
Negative	258 (91.5)	129 (91.5)	129 (91.5)		
Positive	24 (8.5)	12 (8.5)	12 (8.5)		
cT, *n* (%)				0.145	5.393
2	19 (6.7)	9 (6.4)	10 (7.1)		
3	220 (78.0)	109 (77.3)	111 (78.7)		
4a	23 (8.2)	16 (11.3)	7 (5)		
4b	20 ( 7.1)	7 (5)	13 (9.2)		
cN, *n* (%)				0.155	Fisher
0	6 (2.1)	5 (3.5)	1 (0.7)		
1	100 (35.5)	55 (39)	45 (31.9)		
2	139 (49.3)	66 (46.8)	73 (51.8)		
3	37 (13.1)	15 (10.6)	22 (15.6)		
ypT, *n* (%)				0.338	Fisher
0	113 (40.1)	48 (34)	65 (46.1)		
1	36 (12.8)	19 (13.5)	17 (12.1)		
2	56 (19.9)	31 (22)	25 (17.7)		
3	74 (26.2)	41 (29.1)	33 (23.4)		
4	3 (1.1)	2 (1.4)	1 (0.7)		
ypN, *n* (%)				0.969	Fisher
1	188 (66.7)	93 (66)	95 (67.4)		
2	68 (24.1)	34 (24.1)	34 (24.1)		
3	24 (8.5)	13 (9.2)	11 (7.8)		
4	2 (0.7)	1 (0.7)	1 (0.7)		
AJCC/TNM stage, *n* (%)				0.236	Fisher
0	90 (31.9)	37 (26.2)	53 (37.6)		
I	63 (22.3)	36 (25.5)	27 (19.1)		
II	34 (12.1)	19 (13.5)	15 (10.6)		
III	91 (32.3)	46 (32.6)	45 (31.9)		
IV	4 (1.4)	3 (2.1)	1 (0.7)		
pCR, *n* (%)				**0.041**	4.178
Yes	90 (31.9)	37 (26.2)	53 (37.6)		
No	192 (68.1)	104 (73.8)	88 (62.4)		
PT–TRG, *n* (%)				0.135	5.558
0	112 (39.7)	48 (34)	64 (45.4)		
1	67 (23.8)	33 (23.4)	34 (24.1)		
2	83 (29.4)	47 (33.3)	36 (25.5)		
3	20 (7.1)	13 (9.2)	7 (5)		
LN–TRG, *n* (%)				0.996	0.181
0	170 (60.3)	85 (60.3)	85 (60.3)		
1	20 (7.1)	10 (7.1)	10 (7.1)		
2	17 (6.0)	9 (6.4)	8 (5.7)		
3	21 (7.4)	11 (7.8)	10 (7.1)		
4	54 (19.1)	26 (18.4)	28 (19.9)		
Total carcinoma diameter (lymph node), mean ± SD	0.2 ± 0.4	0.2 ± 0.4	0.2 ± 0.3	0.432	0.619
Number of lymph nodes dissected	18.6 ± 8.3	17.6 ± 7.2	19.5 ± 9.2	0.055	3.703
WBC grade, *n* (%)				0.438	0.602
0–2	196 (69.5)	101 (71.6)	95 (67.4)		
3–4	86 (30.5)	40 (28.4)	46 (32.6)		
Hb grade, *n* (%)				0.247	Fisher
0–2	279 (98.9)	141 (100)	138 (97.9)		
3–4	3 (1.1)	0 (0)	3 (2.1)		
Platelet grade, *n* (%)				0.622	Fisher
0–2	278 (98.6)	138 (97.9)	140 (99.3)		
3–4	4 (1.4)	3 (2.1)	1 (0.7)		
Neutrophil grade, *n* (%)				0.275	1.194
0–2	210 (74.5)	109 (77.3)	101 (71.6)		
3–4	72 (25.5)	32 (22.7)	40 (28.4)		
Myelosuppression grade, *n* (%)				0.254	1.3
0–2	189 (67.0)	99 (70.2)	90 (63.8)		
3–4	93 (33.0)	42 (29.8)	51 (36.2)		

Bold indicates that the difference is statistically significant

Table 2 presents the univariate Cox regression analysis, and shows that differentiation, nerve invasion, vascular invasion, ypT, ypN, AJCC/TNM stage, PT–TRG, LN–TRG, the total diameter of lymph node residual cancer, and platelet grade were significant influencing factors for RFS (*p* < 0.05). And after adding all factors with *p* < 0.1 as covariates for adjustment, multivariable Cox regression analysis showed that pCR and LN–TRG (*p* < 0.05) were independent prognostic factors for RFS (Table 2).

**Table 2:** Univariate and multivariable analysis of recurrence-free survival

	Univariate analysis			Multivariable analysis	
Item	HR (95% CI)	*p*-value (Wald’s test)	*p*-value (LR test)	Adj. HR 95% CI	Adj. *p*-value
Sex: male versus Female	0.67 (0.35, 1.29)	0.233	0.209		
Age: < 65 versus ≥ 65 years	1.12 (0.73, 1.71)	0.615	0.618		
Smoker: yes versus no	1.08 (0.7, 1.68)	0.728	0.727		
Drinker: yes versus no	1.17 (0.75, 1.81)	0.49	0.486		
Location: ref. Up			0.211		
Middle	1.75 (0.88, 3.48)	0.112			
Low	1.37 (0.69, 2.74)	0.368			
Differentiation: ref. well			**< 0.001**	1.4 (0.88~2.24)	0.157
Middle	12.77 (1.76, 92.75)	0.012			
Poor	12.19 (1.68, 88.58)	0.013			
Surgical margin: positive versus negative	1.78 (0.44, 7.24)	0.42	0.460		
Nerve Invasion: positive versus negative	2.22 (1.42, 3.47)	< 0.001	**0.001**	2.05 (0.73~5.81))	0.175
Lymph vascular invasion: positive versus negative	2.75 (1.61, 4.72)	< 0.001	**< 0.001**	2.36 (0.94~5.89)	0.067
cT: ref. 2			0.392		
3	0.81 (0.39, 1.68)	0.567			
4a	0.67 (0.25, 1.78)	0.416			
4b	1.36 (0.55, 3.39)	0.507			
cN: ref. 0			0.288		
1	0.89 (0.27, 2.92)	0.85			
2	0.79 (0.24, 2.55)	0.694			
3	1.43 (0.42, 4.94)	0.57			
ypT: ref. 0			**0.016**	1.03(0.51~2.1)	0.929
1	1.4 (0.76, 2.6)	0.285			
2	0.81 (0.41, 1.57)	0.531			
3	1.9 (1.17, 3.09)	0.009			
4	3.49 (1.06, 11.44)	0.039			
ypN: ref. 0			**< 0.001**	0.81 (0.34~1.93)	0.634
1	1.99 (1.26, 3.14)	0.003			
2	2.86 (1.6, 5.1)	< 0.001			
3	5.47 (0.75, 40)	0.094			
AJCC/TNM stage: ref. 0			**0.002**	1.58 (0.17~14.33)	0.683
I	0.87 (0.46, 1.67)	0.682			
II	1.26 (0.63, 2.54)	0.517			
III	2.27 (1.37, 3.77)	0.002			
IV	3.37 (1.01, 11.27)	0.048			
pCR: yes versus no	1.57 (0.98, 2.51)	0.06	0.051	**0.1 (0.01~0.86)**	**0.036**
PT–TRG: ref. 0			**0.024**	0.73 (0.34~1.6)	0.435
1	0.9966 (0.56, 1.75)	0.991			
2	1.63 (1, 2.66)	0.048			
3	2.55 (1.28, 5.07)	0.008			
LN–TRG: 0–2 versus 3–4	2.97(1.97, 4.48)	< 0.001	**< 0.001**	3.62 (1.03~12.74)	**0.045**
Total carcinoma diameter (lymph node) (cont. var.)	2.71 (1.6~4.58)	< 0.001	**< 0.001**	1.95 (0.82~4.62)	0.129
Number of lymph nodes dissected (cont. var.)	0.9962 (0.9714, 1.0215)	0.765	0.764		
WBC grade: 0–2 versus 3–4	1.15 (0.76, 1.75)	0.514	0.517		
Hb grade: 0–2 versus 3–4	1.99 (0.27, 14.34)	0.497	0.54		
Platelet grade: 0–2 versus 3–4	5.25 (1.92, 14.33)	0.001	**0.01**	2.5 (0.4~15.69)	0.327
Neutrophil grade: 0–2 versus 3–4	1.23 (0.8, 1.89)	0.349	0.355		
Myelosuppression grade: 0–2 versus 3–4	1.11 (0.73, 1.68)	0.617	0.619		

Bold indicates that the difference is statistically significant

**Fig. 1:**
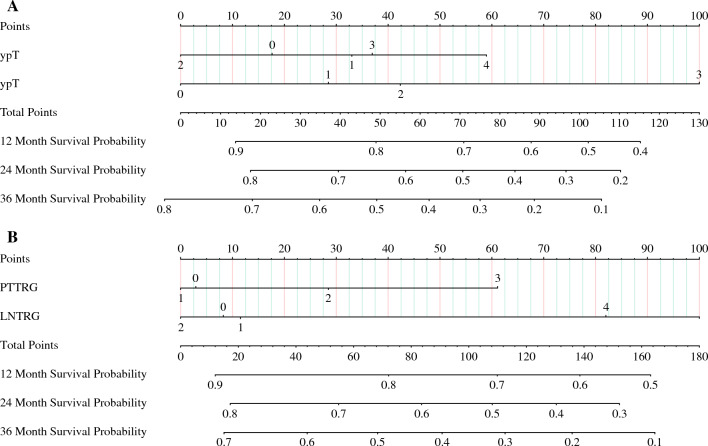
**A** Nomograph of model 1 with ypT and ypN as parameters to predict RFS in patients with esophageal cancer and **B** nomograph of model 2 with PT–TRG and LN–TRG as parameters to predict RFS in patients with esophageal cancer

**Fig. 2:**
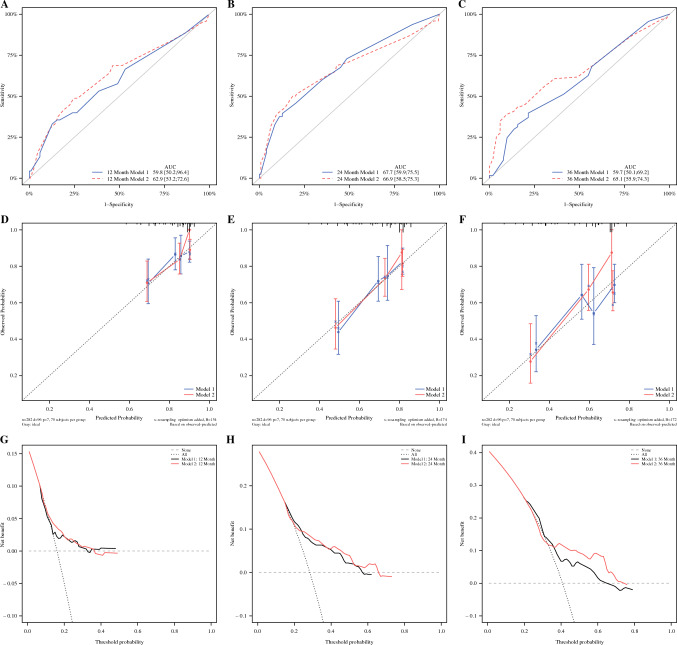
(**A**–**C**) Separation curves of models 1 and 2 at 12, 24, and 36 months; (**D**–**F**) calibration curves of models 1 and 2 at 12, 24, and 36 months; (G–I) decision curves for models 1 and 2 at 12, 24, and 36 months

Figure 1 shows that the model was developed to predict the RFS of patients using ypT, ypN, PT–TRG, and LN–TRG as variables, respectively, and the nomograph of models 1 and 2 was obtained (Fig. 1). The C-indexes of models 1 and 2 were 0.625 (95% CI 0.563~0.686), 0.637 (95% CI 0.575~0.698), the area under the curve (AUC) values for model 1 were 59.8, 67.7, and 59.7 at 12, 24, and 36 months, respectively, and the AUC values for model 2 were 62.9, 66.9, and 65.1 at 12, 24, and 36 months, respectively. Separation curves (Fig. 2A–C), calibration curves (Fig. 2D–F), and decision curves (Fig. 2G–I) are shown for models 1 and 2.

Table 3 demonstrates that, compared with model 1 and model 2, the IDI for predicting RFS at 12, 24, and 36 months was 0.011 (95% CI −0.001 to 0.044), 0.029 (95% CI −0.002 to 0.072), and 0.031 (95% CI −0.002 to 0.083), respectively, with an NRI of 0.165 (95% CI −0.007~0.331), 0.186 (95% CI −0.014~0.399), and 0.119 (95% CI −0.069~0.326), respectively, and model 2 was slightly better than model 1, but not statistically significant (Table 3).

**Table 3:** Model 2 compared with model 1

Model 2 versus model 1	Estimate	Lower 95%	Upper 95%	*p*-value
IDI for 12 month RFS	0.011	−0.01	0.044	0.378
IDI for 24 month RFS	0.029	−0.002	0.072	0.07
IDI for 36 month RFS	0.031	−0.002	0.083	0.06
NRI for 12 month RFS	0.165	−0.007	0.331	0.08
NRI for 24 month RFS	0.186	−0.014	0.399	0.09
NRI for 36 month RFS	0.119	−0.069	0.326	0.209

**Fig. 3:**
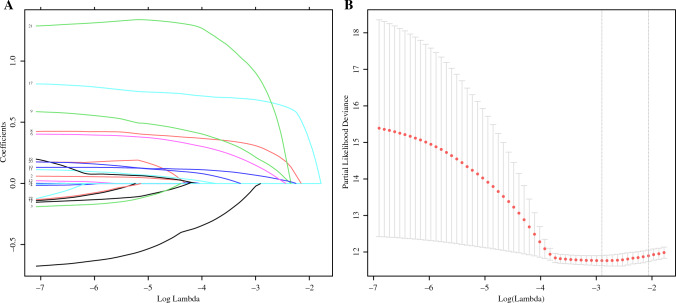
**A** Predictors were selected from 23 clinicopathological factors using the LASSO regression method. **B** When the optimal *λ* was 0.055048, ln(*λ*) = −2.899533, six factors including LN–TRG, vascular invasion, nerve invasion, degree of differentiation, platelet grade, and total diameter of lymph node residual cancer were screened

Figure 3 shows that 23 clinicopathological factors by LASSO regression analysis (AJCC staging, ypT, and ypN staging were not included in the factor screening because of their similar effects) and the *λ* coefficient decreased with the increase in the number of variables, and when the optimal *λ* was 0.055048, ln(*λ*) = −2.899533 (Fig. 3B), the factors whose coefficients were compressed to 0 were excluded, six factors with coefficients that were not 0 were screened out, including LN–TRG, vascular invasion, nerve invasion, degree of differentiation, platelet grade, and total diameter of residual cancer in lymph nodes (Fig. 3A).

**Fig. 4:**
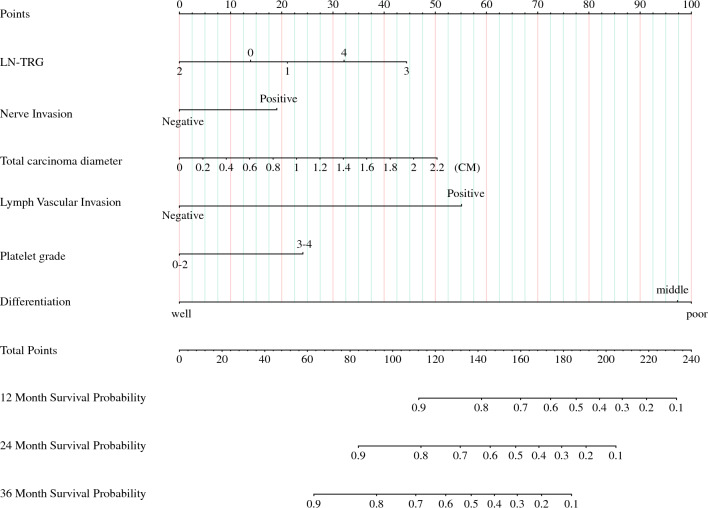
Nomograph of model 3 of six parameters screened by LASSO regression to predict RFS in patients with esophageal cancer

**Fig. 5:**
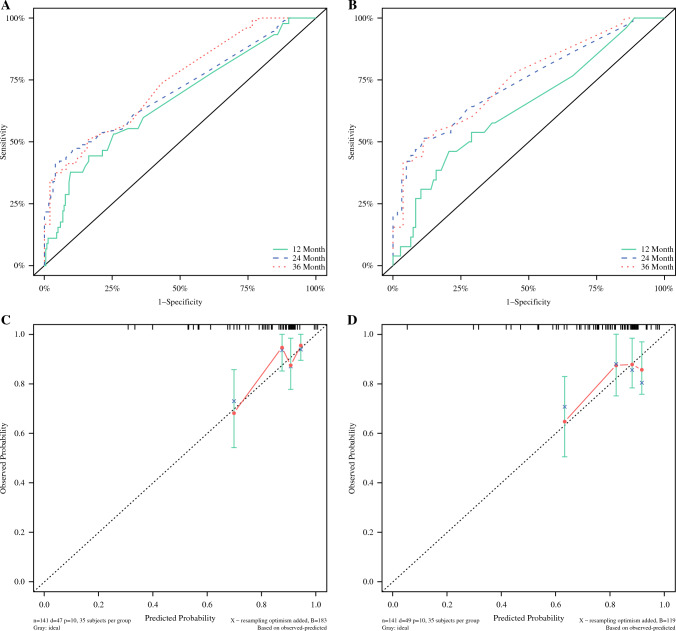

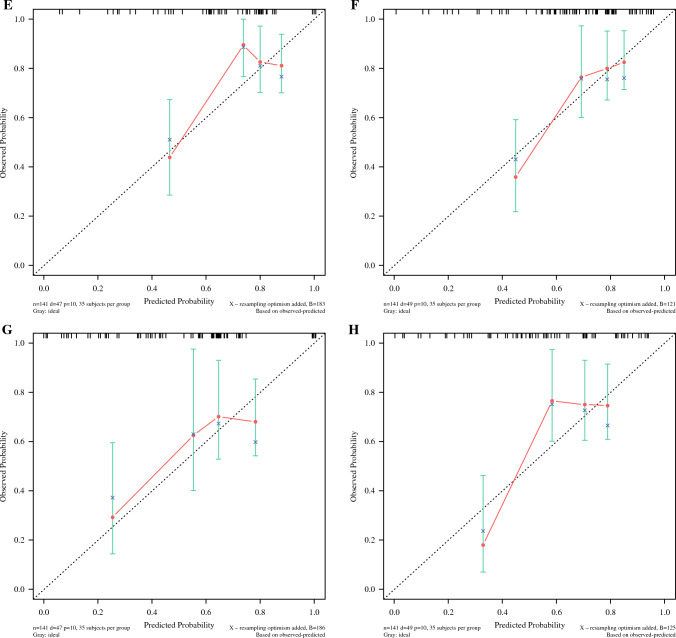
Model 3 separation and calibration curves for the training set and validation set. **A** The separation curve of the training set and **B** the separation curve of the validation. **C**, **E**, **G** Calibration curves for training sets at 12, 24, and 36 months. **D**, **F**, **H** Calibration curves for validation sets at 12, 24, and 36 months

Figure 4 illustrates that the previously screened variables were used to build model 3 for predicting RFS in patients to obtain nomographs (Fig. 4) and for internal validation. The C-index of model 3 was 0.676 (95% CI 0.586~0.765) and 0.681 (95% CI 0.595~0.767) in the training set and validation set, respectively, and the AUC values of model 3 were 73.1, 70.2, and 75.9 at 12, 24, and 36 months in the training set and 63.8, 74.1, and 75.0 at 12, 24, and 36 months in the validation set, respectively, and the separation curves (Fig. 5A,B) and calibration curves (Fig. 5C–H) showed good agreement.

**Fig. 6:**
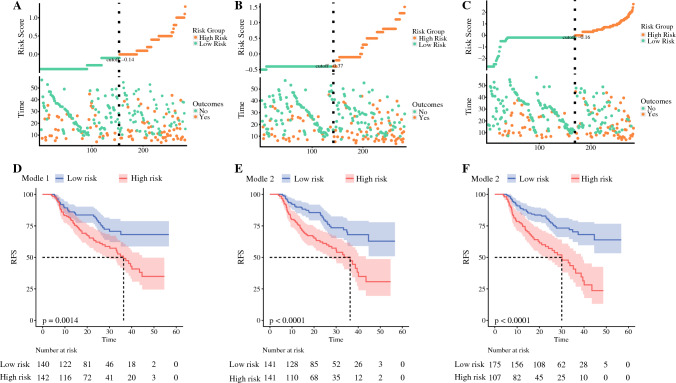
**A**, **B**, **C** Model 1, 2, 3 risk scores, median risk scores, and distribution of survival status. **D**, **E**, **F** Model 1, 2, 3 high- and low-risk K–M curves

Figure 6 shows that, according to the risk coefficients obtained from models 1, 2, and 3, cases were divided into high- and low-recurrence risk groups and K–M curves were plotted, and all models were able to better distinguish between high- and low-risk patients, and models 2 and 3 (*p* < 0.001) had better separation compared with model 1 (*p* = 0.0014) (Fig. 6).

Table 4 shows that, compared with model 2 and model 3, the IDI for predicting RFS at 12, 24, and 36 months was 0.022 (95% CI 0.002~0.09), 0.054 (95% CI 0.014~0.104), and 0.049 (95% CI 0.0~0.094), respectively, with an NRI of 0.67 (95% CI −0.1~0.263), 0.118 (95% CI −0.077~0.268), and 0.087 (95% CI −0.101~0.227), respectively. The IDI of model 3 was superior to model 2 and statistically significant (Table 4).

**Table 4:** Model 3 compared with model 2

Model 3 versus model 2	Estimate	Lower 95%	Upper 95%	*p*-value
IDI for 12 month RFS	0.022	0.002	0.09	**0.03**
IDI for 24 month RFS	0.054	0.014	0.104	**0.01**
IDI for 36 month RFS	0.049	0.0	0.094	**0.05**
NRI for 12 month RFS	0.067	−0.1	0.263	0.348
NRI for 24 month RFS	0.118	−0.077	0.268	0.299
NRI for 36 month RFS	0.087	−0.101	0.227	0.438

Bold indicates that the difference is statistically significant

## Discussion

For patients with locally advanced esophageal squamous carcinoma treated with NCRT combined with surgery, this study is the first to grade the degree of lymph node tumor regression and to develop the first predictive model that includes LN–TRG factors to predict the risk of recurrence. We also verified that the model performs better than AJCC/TMN staging.

The combination of NCRT and surgical treatment has significantly enhanced the prognosis for locally advanced esophageal squamous carcinoma. However, local recurrence and distant metastasis remain significant contributors to poor outcomes. Postoperative adjuvant therapy can improve overall patient prognosis,^8^ but accurately identifying those at high risk for recurrence remains a challenge. Existing studies on the risk of recurrence have focused on the pathological characteristics of the tumor itself, such as the large size of the primary site,^16^ poor differentiation, poor regression, and a high number and proportion of positive lymph nodes^17^ are all independent prognostic factors for recurrence, while some studies have also involved the systemic status of the patient, such as low nutritional index^18^ and alterations in the immunoinflammatory index^13^ are important influencing factors for recurrence. This study collected and analyzed 24 factors including tumor pathological characteristics, hematological characteristics, and basic information. Univariate analysis identified differentiation, nerve invasion, vascular invasion, ypT, ypN, AJCC/TNM stage, PT–TRG, LN–TRG, the total diameter of lymph node residual cancer, and platelet grade as important influencing factors for RFS, while multivariable analysis identified pCR as an independent prognostic factor for RFS, which is in agreement with previous studies. Among them, tumor regression grading, pioneered by us for lymph node assessment, confirmed by multivariable analysis that the independent prognostic factor for RFS is LN–TRG rather than ypN, a finding different from previous studies.

Based on the above findings, we speculate that the comprehensive tumor regression grading for primary site and lymph nodes may have a better predictive effect on tumor recurrence, so in this study, we established model 2 with tumor regression parameters PT–TRG and LN–TRG as variables and model 1 with AJCC/TNM staging parameters ypT and ypN as variables. Both models have a certain degree of discrimination and accuracy, and both can better distinguish between high- and low-risk populations. By comparing the two models through NRI and IDI indices, we found that model 2 is slightly better than model 1 in predictive ability, but this advantage is not obvious. In addition, the LN–TRG factors in both models carried a higher scoring weight, indicating the need for greater attention to be paid to lymph node status. We observed that PT–TRG, LN–TRG, and ypT factors exhibited higher scores in certain lower grades within the model, which could potentially be attributed to variances in pathologists’ subjective assessments. To some extent, the model’s efficacy is constrained; however, incorporating or adjusting additional factors may enhance its potency.

To improve model efficacy, a LASSO regression analysis was used to screen 6 out of 23 factors that were most associated with recurrence, with the following weights in descending order: tumor differentiation, vascular invasion, lymph node residual cancer diameter, LN–TRG, platelet grade, and nerve invasion. (1) The association of poorly differentiated tumors with a recurrence has been demonstrated in a large number of studies,^17^ while He et al.^19^ demonstrated an association between moderately differentiated tumors and NCRT treatment with poor outcomes, and likewise moderately and poorly differentiated tumors in this model were assigned a close and higher score. (2) It is widely recognized that vascular invasion is closely associated with recurrence,^20^ and it is usually theorized that vascular invasion is a prerequisite for lymph node metastasis, which occurred in 83.3% of cases with vascular invasion in this study, while only 21.3% of cases with lymph node metastasis had observed vascular invasion. This differs from expectations, possibly because pathological sections are by nature sampled and the available observed vascular invasion may be predictive of a greater risk of recurrence. (3) The total residual lymph node cancer diameter was calculated by summing the longest diameter of all lymph node live tumor extent to assess residual tumor load, Chen et al.^21^ also predicted disease-free survival by distinguishing whether the lymph nodes were isolated tumor cells, micrometastases, or macrometastases, and demonstrated that macrometastases with greater tumor load were associated with a higher risk of recurrence. This study also demonstrated that the diameter of residual lymph node cancer is an overall important influence on RFS, and in the model, we could observe that the larger the diameter the higher the risk score. (4) LN–TRG was utilized to evaluate lymph node status in this study. While the strong correlation between tumor regression degree and prognosis at the primary site has been widely acknowledged,^22^ there is currently no universally accepted grading standard for lymph node regression. Therefore, we have developed LN–TRG based on the Becker 4 classification standard.^15^ Wu et al.^23^ proposed that the degree of lymph node regression exhibited a stronger correlation with RFS than the degree of primary site regression. Furthermore, our study identified LN–TRG as an independent prognostic factor for RFS, while PT–TRG did not show such significance. These findings are consistent with previous research. Kadota et al.^22^ concluded that there is a prognostic difference between true negative lymph nodes and negative lymph nodes due to the complete regression of metastatic cancer, and this study set grade 0 as true negative lymph nodes to distinguish it from grade 1 as complete regression. Similar to the findings of Wu et al., lower risk scores were obtained for lower grades (grades 0–2, high regression rate) and higher risk scores were obtained for higher grades (grades 3–4, low regression rate), showing that the degree of lymph node regression was associated with RFS. However, the distinction between grades 0, 1, and 2 did not reach the same consistency as in the study of Kadota, T. So, the two-category (high and low) method may exhibit greater consistency than the five-category (0–5) method. (5) For platelet grade, we used the CTCAE 4.03 criteria, which are used more for the assessment of clinical adverse events, and in this study, we found that the degree of platelet reduction during treatment correlated with RFS, which is generally consistent with the findings of Hu et al.^14^ and Cong et al.^24^ Platelet depletion can be used to induce the formation of vascular mimics, which contribute to tumor metastasis,^25^ while platelet adhesion protects tumor cells from immune surveillance and mediates tumor metastasis,^26^ and the depletion of platelets by the aforementioned mechanisms may explain the decrease in platelets in this study.^27^ (6) Nerve invasion accounts for the least weight in the model, Xu et al.^28^ confirmed that the presence of neural invasion is closely associated with prognosis. Furthermore, encapsulated infiltration and penetrating infiltration exhibit distinct prognoses, with penetrating infiltration indicating a more aggressive tumor and a worse prognosis.^29^ Therefore, it is imperative to not only focus on the mere existence or absence of nerve invasion but also pay attention to its infiltration pattern.

There is no predictive model to assess the risk of recurrence of NCRT combined with surgery for locally advanced ESCC, and this study fills this gap. Model 2 incorporates two factors and is easy to operate clinically but has an average evaluation efficacy. In contrast, model 3 incorporated the above-mentioned factors including clinical and pathological dimensions, and obtained consistent and more reliable calibration and separation through internal validation. However, this study also had obvious shortcomings; the single-center study resulted in no external validation and the LN–TRG was not widely accepted and validated. In the future, when we can effectively identify patients at high risk of recurrence, local radiotherapy, chemotherapy, and immunotherapy may be used as postoperative adjuvant therapy to further improve patient survival. However, in the current era, we still have very limited options for adjuvant therapy in high-risk patients.

In summary, our findings validate the superior efficacy of model 3 compared with AJCC/TMN staging and suggest its potential as a complementary tool to the AJCC/TNM staging system. Furthermore, this study provides a research foundation for early adjuvant therapy in high-risk patients; however, additional data collection is required for further validation.

### Supplementary Information

Below is the link to the electronic supplementary material.

Supplementary file1 (DOCX 125 kb)

Supplementary file2 (DOCX 13 kb)

## Data Availability

The data that support the findings of this study are available from the corresponding author upon reasonable request.
