# Supplementary material for: Development and Validation of a Recurrence-Free Survival Prediction Model for Locally Advanced Esophageal Squamous Cell Carcinoma with Neoadjuvant Chemoradiotherapy
Source: Ann Surg Oncol. 2023 Sep 26;31(1):178–91. doi: 10.1245/s10434-023-14308-3 (PMC10695895; doi:10.1245/s10434-023-14308-3)
Supplement: Supplementary file 1 — Supplementary file1 (DOCX 125 kb) [file 10434_2023_14308_MOESM1_ESM.docx]

**
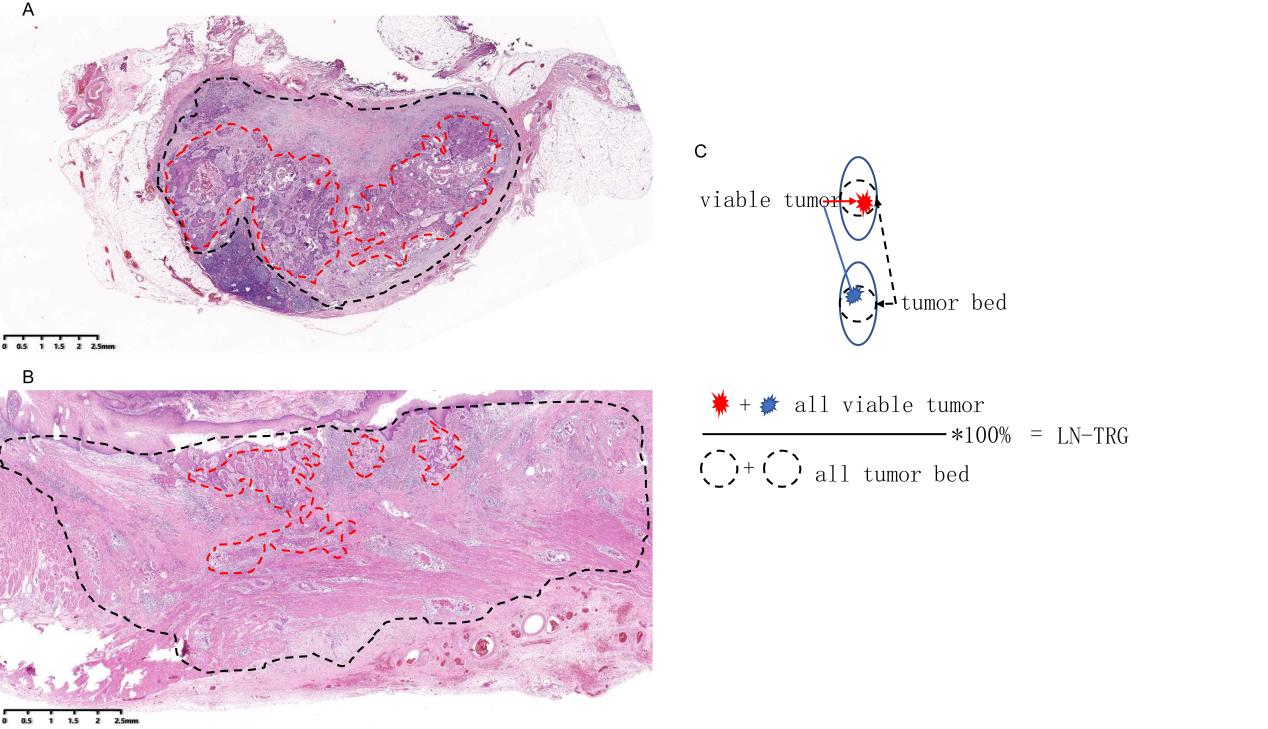
**

**Supplementary Figure 1.** (A) Lymph node regression hematoxylin and eosin (HE) (B) Primary tumor regression hematoxylin and eosin (HE) (C) TRG calculation schematic
