# Supplementary material for: Development and Validation of a Recurrence-Free Survival Prediction Model for Locally Advanced Esophageal Squamous Cell Carcinoma with Neoadjuvant Chemoradiotherapy
Source: Ann Surg Oncol. 2023 Sep 26;31(1):178–91. doi: 10.1245/s10434-023-14308-3 (PMC10695895; doi:10.1245/s10434-023-14308-3)
Supplement: Supplementary file 2 — Supplementary file2 (DOCX 13 kb) [file 10434_2023_14308_MOESM2_ESM.docx]

Supplementary Table 1. Common Terminology Criteria Adverse Events, CTCAE4.03

|  | Grade 1 | Grade 2 | Grade 3 | Grade 4 |
| --- | --- | --- | --- | --- |
| WBC Grade | <LLN-3000/mm3;<LLN-3.0x10e9/L | <3000-2000/mm3<3.0-2.0x10e9/L | <2000-1000/mm3<20-1.0x10e9/L | <1000/mm3;<1.0x10e9/L |
| Hb Grade | Increase in >0-2 gm/dL above ULN or above baseline if the baseline is above ULN | Increase in >2-4gm/dL above ULN or above baseline if the baseline is above ULN | Increase in >4 gm/dL above ULN or above baseline if the baseline is above ULN | - |
| Platelet Grade | <LLN-75000/mm3<LLN-75.0x10e9/L | <75000-50000/mm<750-500x10e9/ | <50000-25000/mm<500-250x10e9/ | <25000/mm<25.0x10e9/L |
| Neutrophil Grade | <LLN-1500/mm3;<LLN-1.5x10e9/L | <1500-1000/mm3;<15-1.0x10e9/ | <1000-500/mm3;<1.0-0.5x10e9/L | <500/mm3;<0.5x10e9/L |
| Myelosuppression Grade | Mildly hypocellular or<=25%reductionfrom normal cellularity for age | Moderately hypocellular  >25-<50% reduction from normal cellularity for age | Severely hypocellular  >50-<=75% reduction cellularity from normal for age | Aplastic persistent for longer than 2 weeks |
